# Supplementary material for: Next-generation CRISPR gene-drive systems using Cas12a nuclease
Source: Nat Commun. 2023 Oct 12;14:6388. doi: 10.1038/s41467-023-42183-9 (PMC10567717; doi:10.1038/s41467-023-42183-9)
Supplement: Supplementary file 1 — Supplementary Information [file 41467_2023_42183_MOESM1_ESM.pdf]

Supplementary Information

Next-generation CRISPR gene-drive systems using Cas12a nuclease

Sara Sanz Juste[1], Emily M. Okamoto[2], Christina Nguyen[4], Xuechun Feng[2,3*], Víctor López Del Amo[4*]

[1] Department of Epigenetics & Molecular Carcinogenesis at MD Anderson, The University of Texas MD Anderson Cancer Center, Houston, TX 77054, USA; Center for Cancer Epigenetics, MD Anderson Cancer Center, Houston, TX, USA.

[2] Section of Cell and Developmental Biology, University of California San Diego, La Jolla, CA 92093, USA

[3] Institute of Infectious Diseases, Shenzhen Bay Laboratory, Shenzhen, Guangdong 518106, China

[4] University of Texas Health Science Center, School of Public Health, Department of Epidemiology, Human Genetics, and Environmental Sciences, Center for Infectious Diseases, Houston, TX 77030, USA

*Correspondence: victor.lopezdelamo@uth.tmc.edu (V.L.D.A); michelle626489@gmail.com (X.F)

Supplementary Figure 1 (Fig S1)

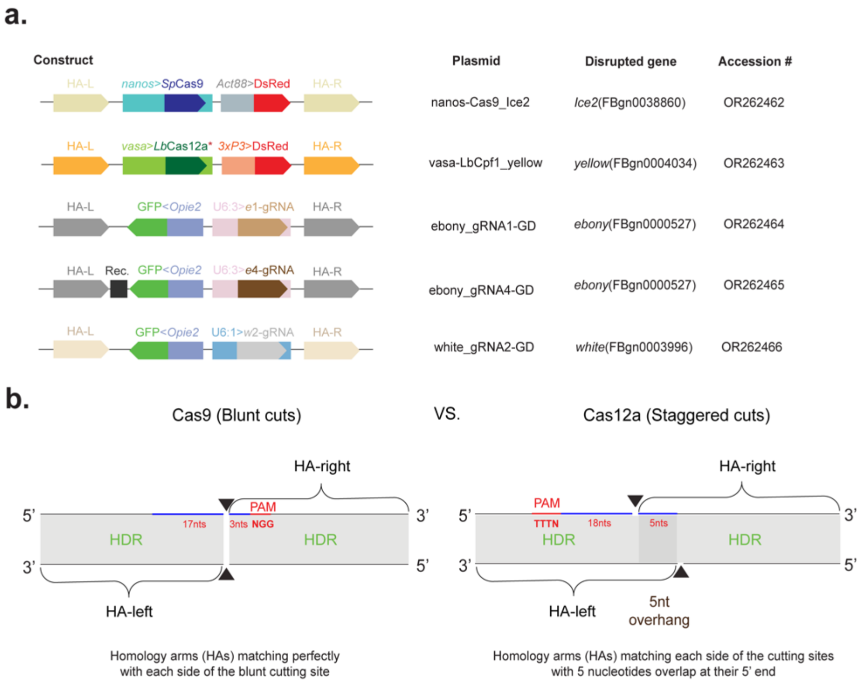

Fig S1 - Related to Figs.1-2 – Gene drive experiments and design. (a) Constructs tested in this work. All transgenes were inserted by HDR and flanked by two homology arms (HA-L and HA-R). Cas9 is driven by the germline-specific *nanos* promoter and marked with DsRed in the eye using the 3xP3 promoter; transgene is inserted into the *ice2* locus. Cas12a is driven by the *vasa* promoter and marked with DsRed in the thorax using the *Opie*2 promoter; transgene is inserted into the *yellow* locus. Gene drive elements (*e1*-gRNA and *e4*-gRNA) are integrated into the *ebony* locus which are driven by the *Drosophila* U6:3 promoter and marked with GFP in the abdomen. The *e4*-GD contains a DNA rescue sequence (Rec.) that restores *ebony* function once the transgene is integrated. The *w2*-GD targeting the *white* gene is marked with GFP in the eye and driven by the *Drosophila* U6:1 promoter. The plasmid names, the genes inserted (disrupted), and the NCBI accession number are listed. **(b)** Homology arms (HA) design differences between Cas9- and Cas12a-based gene drives. The Cas9 nuclease generates blunt cuts and HAs (in light gray) are designed to perfectly match each side of the cut sites. The Cas12a nuclease generates staggered cuts producing a 5nt overhang (darker gray area). Due to the staggered cutting sites generated by the Cas12a nuclease, the 5-nucleotide overhangs were added at the 5' end of each homology arm. The PAM sequences (NGG for Cas9 and TTTN for Cas12a) are highlighted in red color. The gRNAs are highlighted in blue color. Cas9 gRNA length is 20 nucleotides (nts) while Cas12a gRNA length is 23 nts. Black triangles indicate DNA breaks.

Supplementary Figure 2 (Fig S2)

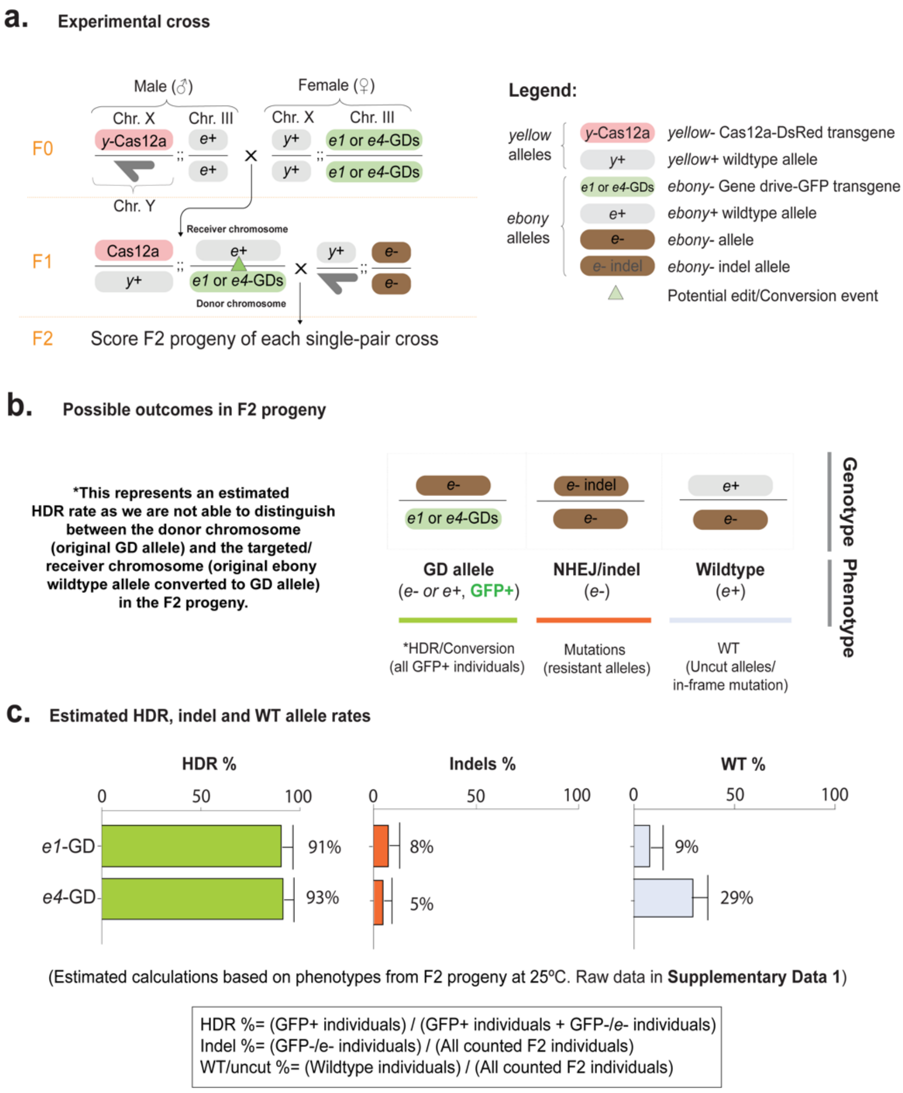

Fig S2 - Related to Fig.1 – Estimated conversion (HDR), indels/resistant alleles and wildtype alleles produced by the Cas12-based CopyCat elements at 25°C. **(a)** Experimental cross with alleles present in our experimental design is depicted. *Yellow* is the chromosome where the Cas12a was inserted. Our gene-drive (GD) elements were inserted in the recessive *ebony* gene. **(b)** The results were graphed according to three possible categories based on the phenotypic readouts: 1) Estimated allelic conversion or HDR (presenting as GFP) 2) indels/resistant allele events – alleles that were cut but were not converted (presenting as GFP-, and ebony phenotype), and 3) wildtype – these individuals displayed wildtype body color, and GFP-, suggesting that these alleles were not acted upon. **(c)** Graphics displaying estimated HDR, Indels and WT alleles rates. HDR: ([all GFP individuals] / [all GFP + indels/mutations]) Indels: ([all GFP + indels] / [all GFP + indels + WT]) WT/uncut alleles: ([WT alleles] / [all GFP + indels + WT]) (see calculation/raw data on **Source Data file 1** for more information).
