# Supplementary material for: Next-generation CRISPR gene-drive systems using Cas12a nuclease
Source: Nat Commun. 2023 Oct 12;14:6388. doi: 10.1038/s41467-023-42183-9 (PMC10567717; doi:10.1038/s41467-023-42183-9)
Supplement: Supplementary file 2 — Reporting Summary [file 41467_2023_42183_MOESM2_ESM.pdf]

nature portfolio

Corresponding author(s): Victor Lopez del Amo and Xuechun Feng

Last updated by author(s): September 25, 2023

Reporting Summary

Nature Portfolio wishes to improve the reproducibility of the work that we publish. This form provides structure for consistency and transparency in reporting. For further information on Nature Portfolio policies, see our Editorial Policies and the Editorial Policy Checklist.

Statistics

For all statistical analyses, confirm that the following items are present in the figure legend, table legend, main text, or Methods section.

n/a	Confirmed	
☐	☒	The exact sample size (*n*) for each experimental group/condition, given as a discrete number and unit of measurement
☐	☒	A statement on whether measurements were taken from distinct samples or whether the same sample was measured repeatedly
☐	☒	The statistical test(s) used AND whether they are one- or two-sided<br>*Only common tests should be described solely by name; describe more complex techniques in the Methods section.*
☒	☐	A description of all covariates tested
☐	☒	A description of any assumptions or corrections, such as tests of normality and adjustment for multiple comparisons
☐	☒	A full description of the statistical parameters including central tendency (e.g. means) or other basic estimates (e.g. regression coefficient) AND variation (e.g. standard deviation) or associated estimates of uncertainty (e.g. confidence intervals)
☐	☒	For null hypothesis testing, the test statistic (e.g. *F*, *t*, *r*) with confidence intervals, effect sizes, degrees of freedom and *P* value noted<br>*Give P values as exact values whenever suitable.*
☒	☐	For Bayesian analysis, information on the choice of priors and Markov chain Monte Carlo settings
☒	☐	For hierarchical and complex designs, identification of the appropriate level for tests and full reporting of outcomes
☒	☐	Estimates of effect sizes (e.g. Cohen's *d*, Pearson's *r*), indicating how they were calculated

Our web collection on statistics for biologists contains articles on many of the points above.

Software and code

Policy information about availability of computer code

Data collection	We did not use any software for data collection
Data analysis	Statistical analysis were performed using Prism 9.0

For manuscripts utilizing custom algorithms or software that are central to the research but not yet described in published literature, software must be made available to editors and reviewers. We strongly encourage code deposition in a community repository (e.g. GitHub). See the Nature Portfolio guidelines for submitting code & software for further information.

Data

Policy information about availability of data

All manuscripts must include a data availability statement. This statement should provide the following information, where applicable:

- Accession codes, unique identifiers, or web links for publicly available datasets
- A description of any restrictions on data availability
- For clinical datasets or third party data, please ensure that the statement adheres to our policy

DNA plasmid sequences are available in https://www.ncbi.nlm.nih.gov/genbank/, and Accession numbers are: OR262462, OR26246 3, OR262464, OR262465, OR262466

Research involving human participants, their data, or biological material

Policy information about studies with human participants or human data. See also policy information about sex, gender (identity/presentation), and sexual orientation and race, ethnicity and racism.

Reporting on sex and gender	N/A
Reporting on race, ethnicity, or other socially relevant groupings	N/A
Population characteristics	N/A
Recruitment	N/A
Ethics oversight	N/A

Note that full information on the approval of the study protocol must also be provided in the manuscript.

Field-specific reporting

Please select the one below that is the best fit for your research. If you are not sure, read the appropriate sections before making your selection.

[x] Life sciences [ ] Behavioural & social sciences [ ] Ecological, evolutionary & environmental sciences

For a reference copy of the document with all sections, see nature.com/documents/nr-reporting-summary-flat.pdf

Life sciences study design

All studies must disclose on these points even when the disclosure is negative.

Sample size	We performed single-pair crosses for all our gene drive experiments displayed in Figures 1 and 2. We obtained at least a number size of >8, which is representative of the overall gene drive performance.
Data exclusions	All raw data provided was included in the figures. Fruit fly crosses with no progeny due to contamination or other causes, were removed from the analysis and are not reported in the raw data tables.
Replication	All the drive inheritance experiments performed include multiple independent technical replicates plotted as individual data points. All attempts of replication were successful with a standard deviation shown in the raw data provided as Supplementary tables within the submission package.
Randomization	F1 female flies, where gene drive activity occurs, were randomly collected from at least 3 different FO crosses.
Blinding	For all the experiments performed we have analyzed fluorescence presence in the eyes or abdomen of fruit flies. This type of scoring does not need the investigators to be blind as the evaluation of the phenotype is presence or absence of the fluorescent marker and there is no much room for interpretation that could be subjective.

Reporting for specific materials, systems and methods

We require information from authors about some types of materials, experimental systems and methods used in many studies. Here, indicate whether each material, system or method listed is relevant to your study. If you are not sure if a list item applies to your research, read the appropriate section before selecting a response.

Materials & experimental systems

n/a	Involved in the study
[x]	[ ] Antibodies
[x]	[ ] Eukaryotic cell lines
[x]	[ ] Palaeontology and archaeology
[ ]	[x] Animals and other organisms
[x]	[ ] Clinical data
[x]	[ ] Dual use research of concern
[x]	[ ] Plants

Methods

n/a	Involved in the study
[x]	[ ] ChIP-seq
[x]	[ ] Flow cytometry
[x]	[ ] MRI-based neuroimaging

Animals and other research organisms

Policy information about studies involving animals; ARRIVE guidelines recommended for reporting animal research, and Sex and Gender in Research

Laboratory animals	All Drosophila melonogaster transgenic animals were generated or assembled in Oregon-R genetic background. Animals age range is 1-15 days.
Wild animals	Study did not involve wild animals.
Reporting on sex	Gene drive allelic conversion was assesed using females (see F1 females in cross schemes, Figures 1 and 2)
Field-collected samples	Study did not involve field-collected samples.
Ethics oversight	Drosophila melonogaster is an invertebrate and is exempt from IACUC oversight.

Note that full information on the approval of the study protocol must also be provided in the manuscript.
